# The association between caffeine intake and risk of kidney stones: A population-based study

**DOI:** 10.3389/fnut.2022.935820

**Published:** 2022-10-10

**Authors:** Jiwen Geng, Yuxuan Qiu, Zhefeng Kang, Yupei Li, Jiameng Li, Ruoxi Liao, Zheng Qin, Qinbo Yang, Baihai Su

**Affiliations:** ^1^Department of Nephrology, West China Hospital, Sichuan University, Chengdu, China; ^2^Department of Ultrasound, West China Hospital, Sichuan University, Chengdu, China; ^3^Department of Cardiovascular Surgery, West China Hospital, Sichuan University, Chengdu, China; ^4^Institute for Disaster Management and Reconstruction, Sichuan University, Chengdu, China; ^5^Department of Nephrology, The First People's Hospital of Shuangliu District, Chengdu, China

**Keywords:** caffeine, kidney stones, coffee, kidney disease, NHANES

## Abstract

**Background:**

Although many studies have proven the beneficial effects of caffeine on human health, the association between caffeine intake and the risk of kidney stones is limited in large epidemiologic studies.

**Objectives:**

We aimed to investigate the association between caffeine intake and the risk of kidney stones.

**Methods:**

A total of 30,716 participants (with weight numbers of 204, 189, and 886) with a history of kidney stone were included in this analysis. All data were survey-weighted, and corresponding logistic regression models were performed to examine the associations between caffeine intake and the risk of kidney stones.

**Results:**

In a fully adjusted model, a per-quartile increase in caffeine intake was associated with a 5.32% decreased risk of kidney stones. In the subgroup analysis, the multivariate-adjusted odds ratios (95% confidence intervals) of the risk of kidney stones for per-quartile increments in caffeine intake were 0.9650 (0.9643, 0.9656) for men, 0.9320 (0.9313, 0.9327) for women, 0.9384 (0.9378, 0.9389) for white race individuals, 1.0281 (1.0270, 1.0292) for nonwhite race individuals, 0.9460 (0.9455, 0.9465) for overweight/obese individuals, and 0.9314 (0.9303, 0.9324) for non-overweight individuals, 0.9100 (0.9094, 0.9105) for caffeine from coffee, and 1.0021 (1.0013, 1.0029) for caffeine from non-coffee sources.

**Conclusion:**

Caffeine intake was negatively associated with the risk of kidney stones. In subgroup analyses, the negative association of caffeine with kidney stone risk was only found in white individuals. In addition, the decreased risk was found higher in women and non-overweight individuals. Especially for women, white individuals and non-overweight individuals. The protective effect of caffeine intake from coffee on stone formation was more significant than that of caffeine from non-coffee sources.

## Background

Kidney stones are a common public health problem with an increasing prevalence worldwide. According to reports, the prevalence of kidney stones was up to 14.8%, of which the prevalence in men was 10–12% and in women was 5–7% (1–4). This disease not only increases the physical pain of patients but also increases the economic burden on society (5–7).

Caffeine is widely consumed around through foods and beverages. Natural caffeine comes from coffee beans, cocoa beans, tea leaves and kola nuts (8). Foods such as chocolate only contribute small amounts of caffeine to consumption, while beverages, especially coffee, with more caffeine than others, are frequently consumed all over the world (9). Humans have a long history of using caffeine, and nowadays, nearly 89% of the adults in the U.S. consumed caffeine every day (10).

In recent years, an increasing number of studies have proven the beneficial effects of caffeine on human health, including the cardiovascular system, nervous system, urinary system and so on (11–13). In addition, previous studies have suggested an association between diet and kidney stones (14, 15), so we suspect that caffeine intake may have a protective effect on the risk of kidney stones. After reviewing the literature, we found some conflicting evidence (16–18). Considering the conflicting and limited evidence on the association between caffeine intake and the risk of kidney stones and the lack of a nationally representative assessment, we used the continuous National Health and Nutrition Examination Survey (NHANES) 2007–2018 databases, which included 204,189,886 samples (weighted number), representing the U.S. populations to evaluate caffeine intake for the risk of kidney stones.

## Methods

### Data source and participants

The NHANES is a periodic survey performed by the National Center for Health Statistics (NCHS). The whole program was approved by the NCHS Ethics Review Board, and all participants have signed informed consent. The NHANES includes extensive demographic data, physical examinations, laboratory tests, health-related questionnaires, and lists of prescription medications. The data are released in 2-year cycles. Six consecutive NHANES 2-year cycles (2007–2008, 2009–2010, 2011–2012, 2013–2014, 2015–2016, and 2017–2018) are collected, as these cycles specifically inquired about the history of kidney stones. Our population consisted of all adults older than 20 years with a history of passing at least one kidney stone. After selection, the NHANES cycles (2007–2018) covered 59,842 participants, of which 8,321 individuals lacked data of caffeine intake, 20,732 participants were not asked the question “Have you ever had kidney stones?” for they were under 20 years old and 73 individuals didn't respond to the question were excluded. Finally, 30,716 participants were included in this analysis with weight numbers of 204, 189, and 886.

The sample weights are created to account for the complex survey design (including oversampling), survey non-response, and post-stratification in order to ensure that calculated estimates are representative of the U.S. civilian noninstitutionalized population. The two-year sample weights for NHANES 2001–2002 and all subsequent two-year cycles are based on population estimates that incorporate the year 2000 Census counts. NCHS does not construct or include all possible weights for the combinations of multiple two-year cycles in the public release files because it would be impractical to do so. Instead, NCHS supplies analysts with information on how to combine these cycles and construct the appropriate weights. When combining two or more two-year cycles from 2001 to 2002 onward, new multi-year sample weights can be computed by simply dividing the two-year sample weights by the number of two-year cycles in the analysis. The participant Flow Chart was shown in Supplementary Figure 1.

### Exposure and outcomes

All NHANES participants were eligible to participate in two 24-hour dietary recall interviews. The first dietary recall interview was collected in person at a mobile testing center, and the second interview was collected by telephone 3–10 days later. Daily totals of nutrients/food components for all foods were calculated for NHANES data collection using the USDA Dietary Study Food and Nutrition Database, which includes approximately 50 coffee/coffee beverages, 30 teas, and caffeinated and non-caffeinated sodas. Therefore, caffeine intake was estimated based on all caffeinated foods and beverages, including energy drinks. The analysis used the average caffeine intake from two 24-hour recalls. Since coffee provides more than 60% of the caffeine consumed (10), the source of caffeine was categorized as “from coffee” and “from non-coffee sources” for the purposes of this analysis. The main result of the analysis was the answer to the question “Have you ever had kidney stones?” and other questions. We considered any subject who reported passing at least one stone as having a history of symptomatic stones.

### Covariables

Based on a previous study on caffeine intake and kidney stone prevalence (17), the following variables were included as covariates: age, sex, body mass index (BMI), total water consumption, alcohol, total energy intake and dietary and supplemental intakes of calcium, phosphate, sodium, potassium, magnesium and vitamins B6, C and D. The mean intake of the above dietary factors from two 24-hour recalls was used in the present analysis. Race and some socioeconomic characteristics were also associated with the prevalence of kidney stones (2), so socioeconomic characteristics, including education level, marital status, and annual household income, were included in this analysis. In addition, smoking and recreational physical activity were included in this analysis.

### Missing covariables

After excluding samples missing data about caffeine intake or history of kidney stone, addresses for 10.2% (3,108 out of 30,716) of the participants could not be geocoded and contributed to missing data in cross-sectional analyses. As such, 10 multiple imputations using fully conditional specifications were used to address potential biases arising from item nonresponse.

### Statistical analysis

All statistical analyses were conducted according to CDC guidelines (https://wwwn.cdc.gov/nchs/nhanes/tutorials/default.aspx). Sample weight was taken into consideration and assigned to each participant (19). Continuous variables are presented as the mean ± standard deviation (SD). Categorical variables are presented as frequencies or percentages. Weighted analysis of variance (ANOVA; for continuous variables) or the chi-square test (for categorical variables) were used to calculate the differences among different groups. Characteristics with three or more categories were treated as indicator (dummy) variables in the logistic analysis.

Weighted logistic regression was used to calculate odds ratios (ORs) and 95% confidence intervals (CIs) for recurrent kidney stones for each quartile of caffeine intake, and we calculated three different logistic regression models. Model 1 was the crude model, and model 2 was adjusted for age (continuous) and sex. Model 3 included the covariates of model 2 with additional adjustment for BMI (continuous), race (Mexican–American, Other Hispanic, non-Hispanic white, non-Hispanic white, Other Race—Including Multi Racial), educational level (< 9th grade, 9–11th grade, high school graduate/GED or equivalent, some college or AA degree, college graduate or above), marital status (married and living with partner or widowed, divorced, separated, and never married), income ratio to poverty (< 1, 1–5 or > 5), total water drank (continuous), smoking (yes, no), vigorous and moderate recreational physical activity for at least 10 min continuously per week (yes, no), and total intakes (continuous) of energy, calcium, phosphate, sodium, potassium, magnesium, alcohol, and vitamins B6, C, and D. Caffeine and dietary confounders (i.e., minerals and vitamins) were adjusted for total energy intake with a residual model, and all of the nutrients were standardized to 2000 kcal (20). Subgroup analysis was performed by gender (men or women), race (white or non-white), BMI (overweight/obese or non-overweight), and caffeine sources (from coffee or from other sources). Weighted stratified line regression models were used to performed subgroup analysis and Interaction terms were used between subgroup indicators to test the effect modification in subgroup.

A value of *p* < 0.05 was considered significant. All analyses were conducted using StataSE 15.1 (StataCorp LLC, College Station).

## Results

### Baseline characteristics of participants

The population characteristics and other covariates of the weighted distribution of included participants in accordance with caffeine intake quartiles are shown in Table 1. The average age of the participants was 47.47 ± 16.88 years; 48.1% of them were men. The weighted rate of kidney stones was 9.95%. The average caffeine intake of the overall participants was 167.91 ± 188.57 mg/day, and the averages of caffeine intake for quartiles 1–4 were 11.28 ± 12.57, 79.22 ± 23.25, 173.14 ± 32.45, and 409.1 ± 223.5, respectively.

**Table 1:** Baseline characteristics of participants.

		**Caffeine intake**				***P*** **-value**
	**Overall**	**Quartile 1**	**Quartile 2**	**Quartile 3**	**Quartile 4**	
		**≤39.5**	**39.5–121.5**	**121.5–237**	**>237**	
Caffeine, mg/day [M (SD)]	167.91 (188.57)	11.28 (12.57)	79.22 (23.25)	173.14 (32.45)	409.01 (223.5)	<0.001
Rate of kidney stones (%)	9.95	9.20	9.83	9.70	11.06	<0.001
Men (%)	48.1	43.94	44.33	47.10	57.05	<0.001
Age [years, M (SD)]	47.47 (16.88)	44.73 (18.31)	45.86 (17.37)	48.79 (16.61)	50.52 (14.32)	<0.001
Race (%)						<0.001
Mexican American	8.38	10.94	11.76	7.32	3.48	
Other Hispanic	5.72	6.8	7.79	5.45	2.85	
Non-Hispanic white	67.02	51.02	57.92	73.64	85.63	
Non-Hispanic black	11.24	22.23	12.86	7.00	2.8	
Other Race	7.63	9.02	9.66	6.59	5.24	
Education (%)						<0.001
<9th grade	5.12	6.71	6.91	4.01	2.85	
9–11th grade	10.35	12.1	10.69	9.00	9.6	
High school graduate	23.09	22.93	23.55	23.00	22.89	
Some college or AA degree	31.47	31.89	30.94	30.04	33.01	
College graduate or above	29.96	26.36	27.92	33.95	31.66	
Married (%)	63.48	56.97	60.27	66.96	69.79	<0.001
Ratio of family income to poverty (%)						<0.001
<1	14.61	19.82	16.97	11.08	10.69	
1–5	59.14	60.29	59.99	58.84	57.50	
>5	26.25	19.88	23.04	30.09	31.81	
BMI (%)						<0.001
<20	4.64	5.13	5.11	4.97	3.36	
20–25	24.69	26.84	24.07	25.14	22.74	
25–30	32.73	30.24	33.07	33.34	34.27	
>30	37.93	37.78	37.74	36.56	39.63	
Vigorous/moderate recreational activities	45.8	42.31	43.33	46.20	51.38	<0.001
for at least 10 min continuously per week (%)						
Smoked at least 100 cigarettes in life (%)	44.13	31.17	37.77	46.72	60.95	<0.001
Daily intake [M (SD)]						
Total energy (kcal)	2,101.78 (854.97)	1,954.77 (849.39)	2,042.87 (822.53)	2,106.28 (792.48)	2,304.11 (911.91)	<0.001
Total water drank (g)	922.25 (921.67)	1,117.72 (1,010.9)	901.95 (938.4)	818.71 (806.2)	850.63 (931.18)	<0.001
Alcohol (gm)	9.7 (23.03)	7.9 (22.95)	8.72 (22.21)	10.42 (21.92)	11.78 (24.72)	<0.001
Vitamin B6 (mg)	2.13 (1.48)	1.99 (1.08)	2.05 (1.23)	2.14 (1.36)	2.36 (2.05)	<0.001
Vitamin C (mg)	81.48 (78.59)	94.69 (88.71)	81.99 (75.62)	78.56 (80.08)	70.6 (66.28)	<0.001
Vitamin D (μg)	4.64 (4.59)	4.75 (4.78)	4.53 (4.38)	4.58 (4.64)	4.68 (4.55)	<0.001
Calcium (mg)	963.71 (515.89)	955.52 (533.57)	936.47 (509.89)	955.89 (498.68)	1,007.03 (518.02)	<0.001
Phosphate (mg)	1,378.30 (591.28)	1,308.11 (601.91)	1,332.39 (569.63)	1,381.83 (554.42)	1,491.34 (619.93)	<0.001
Magnesium (mg)	301.72 (134.11)	287.3 (139.12)	284.17 (127.00)	300.24 (124.38)	335.27 (129.03)	<0.001
Sodium (mg)	3,486.82 (1,539.03)	3,300.36 (1,534.07)	3,379.21 (1,493.29)	3,506.58 (1,480.96)	3,762.37 (1,604.67)	<0.001
Potassium (mg)	2,671.06 (1,108.51)	2,470.30 (1,132.72)	2,492.80 (1,029.46)	2,671.04 (1,075.62)	3,049.50 (1,095.63)	<0.001

Caffeine and dietary confounders (e.g. minerals and vitamins) were adjusted for total energy intake with residual model.

*M*, mean values; SD, standard deviation.

Compared to Quartiles 1–3, participants in Quartile 4 had a higher rate of kidney stones (11.06%, *p* < 0.001). In addition, participants in Quartile 4 were older, often men, more likely to engage in vigorous/moderate recreational activities and smoke. In terms of daily intake, participants in Quartile 4 had higher intake of energy, alcohol, vitamin B6, calcium, phosphate, magnesium, sodium and potassium and lower intake of vitamin C.

### Association between caffeine intake and the risk of kidney stones

The association between caffeine intake and the risk of kidney stones is shown in Table 2. In model 1, a crude model, the risk of kidney stones positively correlated with caffeine intake. The quartile analysis suggested that a per-quartile increment of caffeine intake was associated with a 6.28% increased risk of kidney stones. In model 2, which was adjusted for age and sex, the positive association between them exhibited a weakening trend.

**Table 2:** Association between caffeine intake and kidney stones.

**Caffeine intake**	**OR (95% CI)**, ***P*****-value**		
	**Model 1**	**Model 2**	**Model 3**
Quartile 1			
≤39.5	1.0000 (reference)	1.0000 (reference)	1.0000 (reference)
Quartile 2			
39.5–121.5	1.0755 (1.0741,1 .0769), <0.0001	1.0559 (1.0544, 1.0573), <0.0001	0.9886 (0.9872, 0.9900), <0.0001
Quartile 3			
121–237	1.0597 (1.0583, 1.0611), 0.077	0.9671 (0.9658, 0.9684), <0.0001	0.8612 (0.8599, 0.8625), <0.0001
Quartile 4			
>237	1.2275 (1.2259, 1.2291), <0.0001	1.0610 (1.0596, 1.0624), <0.0001	0.8723 (0.8710, 0.8737), <0.0001
Per quarter	1.0628 (1.0624, 1.0633), <0.0001	1.0097 (1.0093, 1.0101), <0.0001	0.9468 (0.9464, 0.9573), <0.0001
P trend	<0.0001	<0.0001	<0.0001

Caffeine and dietary confounders (e.g. minerals and vitamins) were adjusted for total energy intake with residual model.

Model 1: crude model.

Model 2: adjusted for age and sex.

Model 3: adjusted for age, sex, BMI, educational level, family income, marital status, race, smoking, vigorous and moderate recreational physical activity, total water drank, energy, alcohol, total intakes (from dietary and supplements) vitamins B6, vitamins C, vitamins D, calcium, phosphate, magnesium, sodium, and potassium.

However, in model 3, the model adjusted for all relative covariates, including age, sex, BMI, educational level, family income, marital status, race, smoking, vigorous and moderate recreational physical activity, total water drank, energy, alcohol, total intakes (from dietary and supplements) of vitamins B6, vitamins C, vitamins D, calcium, phosphate, magnesium, sodium, and potassium, and the risk of kidney stones appeared to be negatively correlated with caffeine intake. In models 3, the ORs (95% CIs) of the risk kidney stones for quartile 4 vs. quartile 1 was 0.8723 (0.8710, 0.8737), and per-quartile increments of caffeine intake was associated with 5.32% decreased risk of kidney stones.

### Subgroup analyses

**Figure 1:**
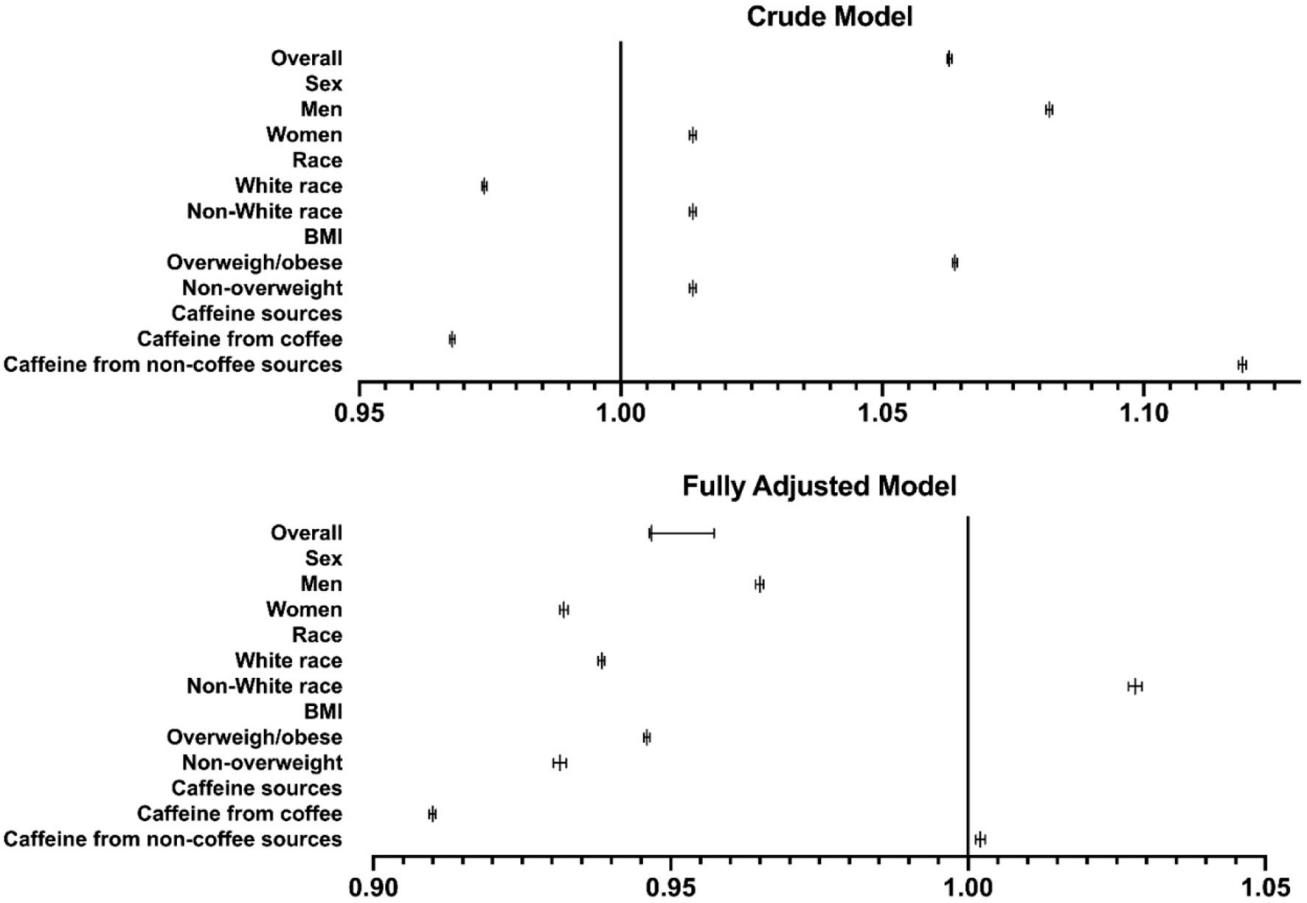
Subgroup analysis. ORs for the association between caffeine intake and kidney stone. The subgroup analyses results of crude model (model 1) and fully adjusted model (model 3). Abscissa represents odds ratios (ORs) and 95% CI (confidence intervals). The figure showed the OR with 95%CI for the trend of risk of kidney stones as the caffeine intake quarte rise.

To further explore the factors affecting the association between caffeine intake and the risk of kidney stones, we performed stratified analysis by sex, race, BMI, and caffeine sources. Figure 1 present the subgroup analyses results of crude model (model 1) and fully adjusted model (model 3). The detailed analysis results are shown in Supplementary Table 1. In model 3, the fully adjusted model, the ORs (95% CIs) of the risk of kidney stones for quartile 4 vs. quartile 1 were 0.9596 (0.9576, 0.9616) for men and 0.8085 (0.8067, 0.8103) for women, and a per-quartile increment of caffeine intake was associated with a 3.50 and 6.80% decreased risk of kidney stones for men and women, respectively.

In terms of race, in model 3, the ORs (95% CIs) of the risk of kidney stones for quartile 4 vs. quartile 1 were 0.8428 (0.8414, 0.8443) for white race and 1.1786 (1.1745, 1.1827) for nonwhite race. A per-quartile increase in caffeine intake was associated with a 6.16% decreased and 2.81% increased risk of kidney stones for white race and nonwhite race, respectively. The stratified analysis by race suggested that white race was a protective factor in the association between caffeine intake and the risk of kidney stones.

Participants with a BMI of 25 or greater were defined as overweight/obese. The ORs (95% CIs) of the risk of kidney stones for quartile 4 vs. quartile 1 were 0.8686 (0.8671, 0.8701) for overweight participants and 0.8168 (0.8140, 0.8197) for non-overweight participants.

Caffeine sources also affected the association between caffeine intake and the risk of kidney stones. The ORs (95% CIs) of the risk of kidney stones for quartile 4 vs. quartile 1 were 0.7706 (0.7692, 0.7721) for caffeine from coffee and 1.0431 (1.0404, 1.0457) for caffeine from noncoffee sources. In addition, a per-quartile increment of caffeine intake from coffee was associated with a 9.00% decreased risk of kidney stones, which suggested that compared with caffeine from non–coffee sources, caffeine from coffee was more advantageous in reducing the risk of kidney stones.

## Discussion

Our research demonstrated a significant negative independent association between caffeine intake and the risk of kidney stones. The negative association of caffeine with kidney stone risk was only found in white individuals. In the opposite, a positive association was found in non-white individuals. Although the association was found in both men and women, the decreased risk was found higher in women. Compared with caffeine from non-coffee sources, caffeine from coffee was more advantageous in reducing the risk of kidney stones. Actually, caffeine from other sources was associated with an increased risk of kidney stones.

The negative association presented a similar trend as a previous study (17). Ferraro's study (17) included 3 cohorts, all of which consisted of white participants, one of whom consisted of men and two of whom consisted of women. Thus, both sex and race were the limitations of their study. By comparison, our analysis was based on the representative sample of the U.S. population, and more comprehensive demographic information was considered, which made up for the limitations of previous research in some ways. Sun (16) also used the NHANES database, and their study demonstrated that caffeine intake was associated with a higher risk of recurrent kidney stones in adults, which might be conflicting with our results. The accuracy of the definition of “recurrent kidney stones” might be the reason. In contrast, in our study we focused on kidney stone risk, including more participants. Similarly, a case–control study with 39 patients suggested a negative association between caffeine intake and the risk of calcium oxalate stone formation (18). Considering the small sample size and recall bias, its results still need to be further explored for validation. Based on the above controversial evidence, our research is necessary and meaningful.

Although the mechanisms underlying caffeine intake and the risk of kidney stones remain unclear, some evidence can support the negative association between them. Caffeine has diuretic properties and can increase the water intake and urine flow rate of the user (21–23). They are all important protective factors against the development of kidney stones. In addition, coffee consumption was thought to be beneficial to kidney function (24), and daily coffee intake could even decrease the risk of the development of chronic kidney disease (25). Calcium kidney stones are the most common type of kidney stones (26). The majority of stones (more than 80%) are composed of calcium oxalate (CaOx) mixed with calcium phosphate (CaP) (4, 27). Previous study also indicated the importance of calcium balance on kidney stones (28). Although caffeine has the ability to increase the urinary excretion of calcium (29, 30), the negative association between caffeine intake and urinary supersaturation of calcium oxalate and uric acid might explain the protective effect of caffeine on kidney stones (17). At the molecular biology level, the protective effect of caffeine is achieved through reducing the crystal-binding capacity of renal tubular epithelial cells (31). Our results also indicated that caffeine from coffee was more advantageous in reducing the risk of kidney stones while caffeine from other sources is associated with an increased risk of kidney stones. Thus, there might be a possible that other molecules in coffee could act synergistically with caffeine to produce protective effects.

Previous studies have shown that sex, race-ethnicity, and obesity are factors affecting the prevalence of kidney stones (2, 32, 33). The source of caffeine is also closely related to the formation of kidney stones (34–36). Thus, in the subgroup analysis, we performed stratified analyses by sex, race-ethnicity, obesity and caffeine sources. It was reported that men are more likely to have kidney stones (2, 32). Similarly, the protective effect of caffeine on the risk of kidney stones is more pronounced in women than men. White individuals were more likely to have a history of stone disease than non-white individuals (2). Interestingly, the stratified analysis by race suggested that individuals of white race are more sensitive to the protective effect of caffeine on kidney stones. In addition, caffeine from coffee was more advantageous in reducing the risk of kidney stones. The stratified analysis by caffeine sources showed that the ORs (95% CIs) of the risk of kidney stones for quartile 4 vs. quartile 1 were 0.7706 (0.7692, 0.7721) for caffeine from coffee, while the risk reduction was not obvious for caffeine from non-coffee sources. It was reported that decaffeinated coffee could also decrease the risk of forming stones (17, 34, 35), which suggested that in addition to caffeine, there might be other components in coffee that reduce the risk of stone formation. Thus, coffee consumption is a great idea to reduce the risk of developing kidney stones. Recently, the negative association of caffeine with kidney stone risk has also been confirmed by genetic data (37).

Analyzing a representative sample of the U.S. population is one strength of this study. A total of 204, 189, and 886 samples and standardized protocols of NHANES not only minimized any possible bias but also made our results reliable. To our knowledge, the sample size we included is the largest of the available studies on caffeine intake and the risk of kidney stones. In addition, we adjusted covariates associated with exposure and outcomes to ensure that our results were applicable to a wide range of populations. However, there were still several limitations in this study. First, as a cross-sectional study, the evidence for temporal relations and causal inference was limited. Second, the caffeine intake data were obtained from 24-hour recalls, which might lead to potential intraperson variability and recall bias. Third, due to lack of information on date of kidney stones, there might be a possible that kidney stones occurred before the collection of food intakes, which might add bias to our results. At last, due to the lack of data for kidney stone composition, we cannot further analyze the association between caffeine intake and different types of kidney stones.

In conclusion, our results showed that caffeine intake was negatively associated with the risk of kidney stones. In subgroup analyses, the negative association of caffeine with kidney stone risk was only found in white individuals. In addition, the decreased risk was found higher in women and non-overweight individuals. The protective effect of caffeine intake from coffee for stone formation was more significant than caffeine from noncoffee sources.

## Data availability statement

The original contributions presented in the study are included in the article/Supplementary material, further inquiries can be directed to the corresponding authors.

## Ethics statement

The studies involving human participants were reviewed and approved by the National Center for Health Statistics.

## Author contributions

JG and YQ designed the research, analyzed the data, and wrote the paper. ZK, YL, JL, and RL assisted in the data analysis. ZQ and QY assisted in manuscript preparation. BS had primary responsibility for the final content. All authors read and approved the final manuscript.

## Funding

This work was financially sponsored by the National Natural Science Foundation of China (Grant No. 82000702), the Science and Technology Achievement Transformation Fund of West China Hospital of Sichuan University (Grant No. CGZH19006), the 1.3.5 project for disciplines of excellence from West China Hospital of Sichuan University (Grant No. ZYJC21010), and Med+ Biomaterial Institute of West China Hospital/West China School of Medicine of Sichuan University (Grant No. ZYME20001).

## Conflict of interest

The authors declare that the research was conducted in the absence of any commercial or financial relationships that could be construed as a potential conflict of interest.

## Publisher's note

All claims expressed in this article are solely those of the authors and do not necessarily represent those of their affiliated organizations, or those of the publisher, the editors and the reviewers. Any product that may be evaluated in this article, or claim that may be made by its manufacturer, is not guaranteed or endorsed by the publisher.
