# Supplementary material for: The association between caffeine intake and risk of kidney stones: A population-based study
Source: Front Nutr. 2022 Oct 10;9:935820. doi: 10.3389/fnut.2022.935820 (PMC9589282; doi:10.3389/fnut.2022.935820)
Supplement: Supplementary file 1 [file Table_1.docx]

**Supplemental Table 1.** **Subgroup analysis.**

|  | OR (95% CI), P value | | | | | |
| --- | --- | --- | --- | --- | --- | --- |
| Caffeine intake | Men | | | Women | | |
|  | Model 1 | Model 2 | Model3 | Model 1 | Model 2 | Model3 |
| Quartile 1  ≤39.5 | 1.0  (reference) | 1.0  (reference) | 1.0  (reference) | 1.0  (reference) | 1.0  (reference) | 1.0  (reference) |
| Quartile 2  39.5 to 121.5 | 1.2362  (1.2338,1.2385)  <0.0001 | 1.1674  (1.1651,1.1697)  <0.0001 | 1.1426  (1.1402,1.1450)  <0.0001 | 0.9404  (0.9387,0.9421)  <0.0001 | 0.8849  (0.8832,0.8865)  <0.0001 | 0.8789  (0.8772,0.8806)  <0.0001 |
| Quartile 3  121.5 to 237 | 1.1296  (1.1274,1.1317)  <0.0001 | 0.8928  (0.8911,0.8946)  <0.0001 | 0.9089  (0.9069,0.9108)  <0.0001 | 0.9872  (0.9854,0.9890)  <0.0001 | 0.8493  (0.8477,0.8509)  <0.0001 | 0.8385  (0.8368,0.8402)  <0.0001 |
| Quartile 4  >237 | 1.3392  (1.3368,1.3416)  <0.0001 | 0.9675  (0.9657,0.9693)  <0.0001 | 0.9596  (0.9576,0.9616)  <0.0001 | 1.0357  (1.0337,1.0377)  <0.0001 | 0.8472  (0.8455,0.8489)  <0.0001 | 0.8085  (0.8067,0.8103)  <0.0001 |
| Per quarter | 1.0819 (1.0813,1.0825)  <0.0001 | 0.9645  (0.9639,0.9650)  <0.0001 | 0.9650  (0.9643,0.9656)  <0.0001 | 1.0138  (1.0131,1.0144)  <0.0001 | 0.9456  (0.9450,0.9462)  <0.0001 | 0.9320  (0.9313,0.9327)  <0.0001 |
| P trend | <0.0001 | <0.0001 | <0.0001 | <0.0001 | <0.0001 | <0.0001 |
| Caffeine intake | White race | | | Non-white race | | |
|  | Model 1 | Model 2 | Model3 | Model 1 | Model 2 | Model3 |
| Quartile 1  ≤39.5 | 1.0  (reference) | 1.0  (reference) | 1.0  (reference) | 1.0  (reference) | 1.0  (reference) | 1.0  (reference) |
| Quartile 2  39.5 to 121.5 | 0.9773  (0.9757,0.9788)  <0.0001 | 0.9864  (0.9848,0.9881)  <0.0001 | 0.9696  (0.9679,0.9713)  <0.0001 | 1.1496  (1.1469,1.1522)  <0.0001 | 1.1031  (1.1006,1.1057)  <0.0001 | 1.1103  (1.1075,1.1131)  <0.0001 |
| Quartile 3  121.5 to 237 | 0.8792  (0.8779,0.8806)  <0.0001 | 0.8540  (0.8527,0.8554)  <0.0001 | 0.8516  (0.8501,0.8530)  <0.0001 | 1.0881  (1.0852,1.0911)  <0.0001 | 0.9870  (0.9843,0.9896)  <0.0001 | 0.9655  (0.9627,0.9684)  <0.0001 |
| Quartile 4  >237 | 0.9390  (0.9376,0.9404)  <0.0001 | 0.9055  (0.9041,0.9069)  <0.0001 | 0.8428  (0.8414,0.8443)  <0.0001 | 1.4957  (1.4912,1.5001)  <0.0001 | 1.2976  (1.2937,1.3016)  <0.0001 | 1.1786  (1.1745,1.1827)  <0.0001 |
| Per quarter | 0.9739  (0.9735,0.9744)  <0.0001 | 0.9594  (0.9590,0.9599)  <0.0001 | 0.9384  (0.9378,0.9389)  <0.0001 | 1.0138  (1.0131,1.0144)  <0.0001 | 1.0566  (1.0556,1.0576)  <0.0001 | 1.0281  (1.0270,1.0292)  <0.0001 |
| P trend | <0.0001 | <0.0001 | <0.0001 | <0.0001 | <0.0001 | <0.0001 |
| Caffeine intake | Overweight/obese | | | Non-overweight | | |
|  | Model 1 | Model 2 | Model3 | Model 1 | Model 2 | Model3 |
| Quartile 1  ≤39.5 | 1.0  (reference) | 1.0  (reference) | 1.0  (reference) | 1.0  (reference) | 1.0  (reference) | 1.0  (reference) |
| Quartile 2  39.5 to 121.5 | 1.0271  (1.0256,1.0287)  <0.0001 | 1.0102  (1.0087,1.0118)  <0.0001 | 0.9554  (0.9538,0.9569)  <0.0001 | 1.2318  (1.2283,1.2354)  <0.0001 | 1.2116  (1.2081,1.2151)  <0.0001 | 1.0928  (1.0895,1.0962)  <0.0001 |
| Quartile 3  121.5 to 237 | 1.0210  (1.0195,1.0226)  <0.0001 | 0.9308  (0.9293,0.9322)  <0.0001 | 0.8290  (0.8276,0.8303)  <0.0001 | 1.2035  (1.2000,1.2069)  <0.0001 | 1.1140  (1.1108,1.1172)  <0.0001 | 0.9568  (0.9539,0.9598)  <0.0001 |
| Quartile 4  >237 | 1.2203  (1.2185,1.2221)  <0.0001 | 1.0689  (1.0673,1.0705)  <0.0001 | 0.8686  (0.8671,0.8701)  <0.0001 | 1.1263  (1.1229,1.1297)  <0.0001 | 0.9752  (0.9722,0.9782)  <0.0001 | 0.8168  (0.8140,0.8197)  <0.0001 |
| Per quarter | 1.0639  (1.0634,1.0644)  <0.0001 | 1.0234  (1.0234,1.0234)  <0.0001 | 0.9460  (0.9455,0.9465)  <0.0001 | 1.0138  (1.0131,1.0144)  <0.0001 | 0.9868  (0.9859,0.9877)  <0.0001 | 0.9314  (0.9303,0.9324)  <0.0001 |
| P trend | <0.0001 | <0.0001 | <0.0001 | <0.0001 | <0.0001 | <0.0001 |
| Caffeine intake | Caffeine from coffee | | | Caffeine from non–coffee sources | | |
|  | Model 1 | Model 2 | Model3 | Model 1 | Model 2 | Model3 |
| Quartile 1  ≤39.5 | 1.0  (reference) | 1.0  (reference) | 1.0  (reference) | 1.0  (reference) | 1.0  (reference) | 1.0  (reference) |
| Quartile 2  39.5 to 121.5 | 1.0094  (1.0077,1.0112)  <0.0001 | 1.0125  (1.0108,1.0143)  <0.0001 | 0.9526  (0.9508,0.9544)  <0.0001 | 1.1457  (1.1433,1.1481)  <0.0001 | 1.1459  (1.1435,1.1483)  <0.0001 | 1.0650  (1.0626,1.0674)  <0.0001 |
| Quartile 3  121.5 to 237 | 0.8811  (0.8796,0.8825)  <0.0001 | 0.8638  (0.8623,0.8653)  <0.0001 | 0.8074  (0.8059,0.8090)  <0.0001 | 1.1753  (1.1727,1.1778)  <0.0001 | 1.1760  (1.1734,1.1785)  <0.0001 | 0.9670  (0.9647,0.9693)  <0.0001 |
| Quartile 4  >237 | 0.9336  (0.9321,.9351)  <0.0001 | 0.8687  (0.8673,0.8701)  <0.0001 | 0.7706  (0.7692,0.7721)  <0.0001 | 1.4444  (1.4412,1.4475)  <0.0001 | 1.4476  (1.4445,1.4508)  <0.0001 | 1.0431  (1.0404,1.0457)  <0.0001 |
| Per quarter | 0.9678  (0.9673,0.9683)  <0.0001 | 0.9442  (0.9437,0.9447)  <0.0001 | 0.9100  (0.9094,0.9105)  <0.0001 | 1.1189  (1.1181,1.1196)  <0.0001 | 1.1196  (1.1188,1.1203)  <0.0001 | 1.0021  (1.0013,1.0029)  <0.0001 |
| P trend | <0.0001 | <0.0001 | <0.0001 | <0.0001 | <0.0001 | <0.0001 |

Caffeine and dietary confounders (e.g. minerals and vitamins) were adjusted for total energy intake with residual model

Model 1: crude model

Model 2: adjusted for age and sex

Model 3: adjusted for age, sex, BMI, educational level, family income, marital status, race, smoking, vigorous and moderate recreational physical activity, total water drank, energy, alcohol, total intakes (from dietary and supplements) vitamins B6, vitamins C, vitamins D, calcium, phosphate, magnesium, sodium, and potassium.

The showing results of subgroup analysis were adjusted for above covariates except effect modifier.
