# Supplementary figures and images for: The association between caffeine intake and risk of kidney stones: A population-based study
Source: Front Nutr. 2022 Oct 10;9:935820. doi: 10.3389/fnut.2022.935820 (PMC9589282; doi:10.3389/fnut.2022.935820)

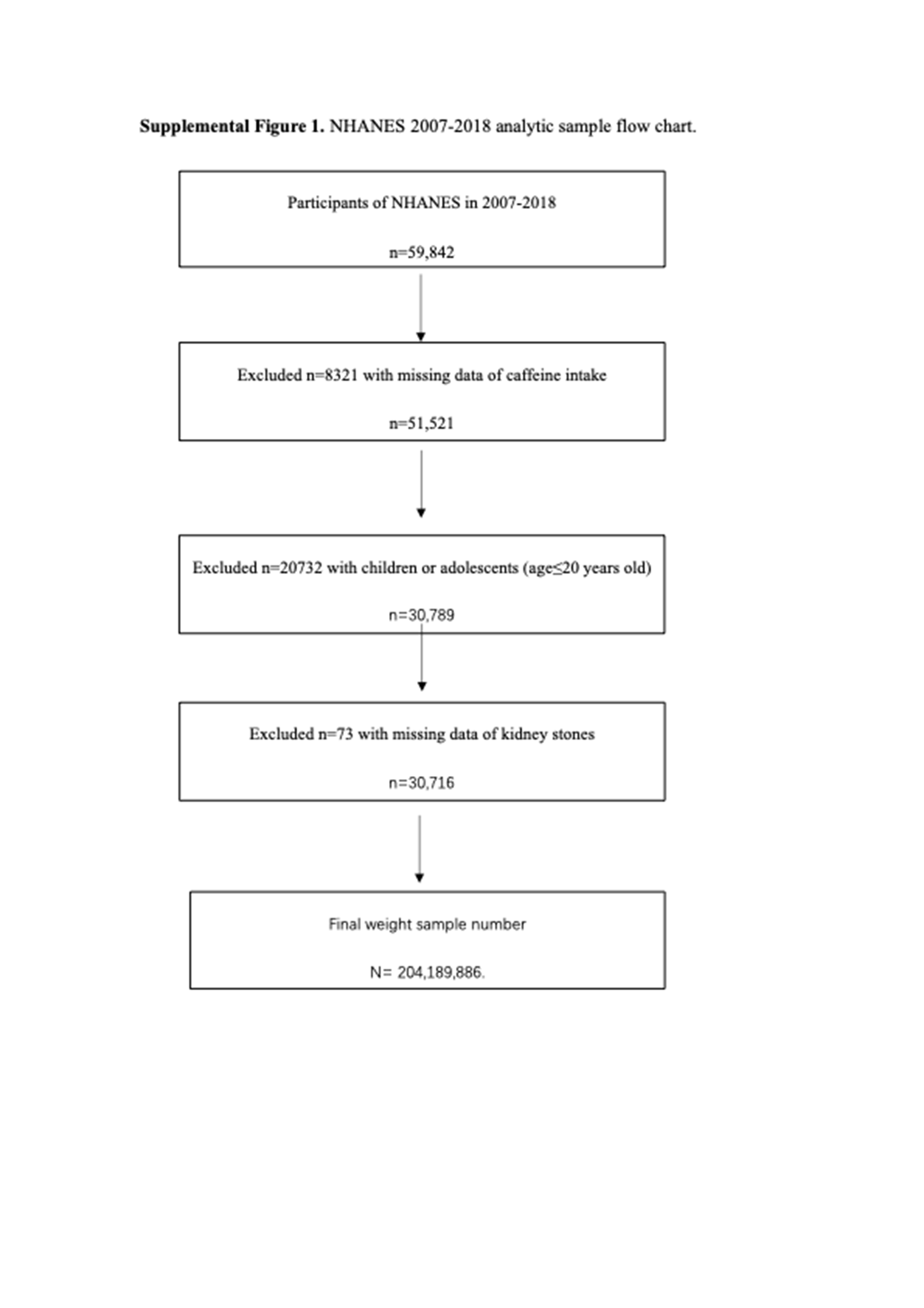

Supplement: Supplementary file 2 [file Image_1.TIFF]
